# Ventilator associated- respiratory infections (VARI), are all the same?

**DOI:** 10.1186/2197-425X-3-S1-A699

**Published:** 2015-10-01

**Authors:** M Palomar, F Alvarez Lerma, S Uriona, X Nuvials, P Olaechea, M Catalan, S Otero, R Gimeno, ENVIN-HELICS

**Affiliations:** Hospital Universitari Arnau de Vilanova, ICU, Lleida, Spain; Hospital Mar, Barcelona, Spain; Hospital Vall Hebron, Barcelona, Spain; Hospital Universitari Arnau de Vilanova, Lleida, Spain; Hospital Mar Galdakao, Bilbao, Spain; Hospital Doce de Octubre, Madrid, Spain; Hospital La Fe, Valencia, Spain

## Introduction

Ventilator associated- respiratory infections (VARI) are the most prevalent in the ICU. The differential diagnosis of pneumonia (VAP) and tracheobronchitis (TAV) is overlapping.

## Objectives

The aim of this study is to analyze the differences in presentation and outcome between VAP and TAV in a large database.

## Methods

Analysis of the Spanish ENVIN register 2008 2014 database. Diagnoses definitions included in the manual (ECDC and CDC). Characteristics of patients with TAV and NAV, of all episodes of VARI and patient outcomes were analyzed. Univariate and multivariate analysis were conducted.

## Results

Among 115 966 patients hospitalized > 24 h, 91,122 (78.6%) with full information were analyzed. The 55% of them (50,212) were ventilated for 498,316 days. In 6699 (7.4% of total and 13.3% of ventilated) 8,131 VARI were diagnosed of which 4,435 (54.5%) were classified as VAP and 3,696 as VAT. In 3,433 pts 1 or more episodes of VAP were identified and TAV in 2,905 pts. Only 361 pts were recorded as having episodes of VAP +VAT. The incidence was 10.9 VAP x 1000 days of MV and 9.1 VAT x 1000 days of MV.

VAP was significantly more frequent in younger patients (p < 0.001), with traumatic, OR 1.3 (1.1-1.5) and medical OR 1.21 (1.1-1.4) pathology and neutropenia OR 1.4 (1.03 to 2.1). No differences in APACHE II at admission, origin of the patients (community, ward, nursing homes), use of devices (CVC, UC etc), emergency surgery, extra renal depuration, immunosuppression or antibiotics at admission or before VARI. Days prior to the VARI diagnosis were 10.3 (8.8) globally, 9.7 for VAP and (8.6) for VAT. However, systemic inflammatory response (RIS) was significantly higher (p < 0.0001) in VAP (severe sepsis 24.2%, septic shock 18.8%) than in VAT (8 and 3.2%). Regarding the impact on outcome, the total duration of MV, 23.7 (15.4) vs. 20.6 (15) days and ICU stay, 27.4 (15.6) vs. 24.5 (15) days were higher in patients with VAP. Likewise, the crude mortality rate was higher in patients with VAP, OR 2.4 (95% CI 2.1 to 2.7), while mortality of patients with VAT was similar to patients without VARI.

## Conclusions

VAT and VAP had a similar incidence, but the presentation of the episodes (RIS) and the impact on the ICU-stay and mortality are clearly different.
